# A Comparative Study of Natural Language Processing Algorithms Based on Cities Changing Diabetes Vulnerability Data

**DOI:** 10.3390/healthcare10061119

**Published:** 2022-06-15

**Authors:** Siting Wang, Fuman Song, Qinqun Qiao, Yuanyuan Liu, Jiageng Chen, Jun Ma

**Affiliations:** School of Public Health, Tianjin Medical University, Qixiangtai Road 22, Heping District, Tianjin 300070, China; wst2016041323@163.com (S.W.); songfm1002@163.com (F.S.); qiaoqinqunqqq@163.com (Q.Q.); ivyuan10@126.com (Y.L.); junma@tmu.edu.cn (J.M.)

**Keywords:** T2DM, NLP, BERT, ERNIE

## Abstract

(1) Background: Poor adherence to management behaviors in Chinese Type 2 diabetes mellitus (T2DM) patients leads to an uncontrolled prognosis of diabetes, which results in significant economic costs for China. It is imperative to quickly locate vulnerability factors in the management behavior of patients with T2DM. (2) Methods: In this study, a thematic analysis of the collected interview materials was conducted to construct the themes of T2DM management vulnerability. We explored the applicability of the pre-trained models based on the evaluation metrics in text classification. (3) Results: We constructed 12 themes of vulnerability related to the health and well-being of people with T2DM in Tianjin. We considered that Bidirectional Encoder Representation from Transformers (BERT) performed better in this Natural Language Processing (NLP) task with a shorter completion time. With the splitting ratio of 6:3:1 and batch size of 64 for BERT, the test accuracy was 97.71%, the completion time was 10 min 24 s, and the macro-F1 score was 0.9752. (4) Conclusions: Our results proved the applicability of NLP techniques in this specific Chinese-language medical environment. We filled the knowledge gap in the application of NLP technologies in diabetes management. Our study provided strong support for using NLP techniques to rapidly locate vulnerability factors in T2DM management.

## 1. Introduction

Type 2 diabetes mellitus (T2DM) has become the most impactful non-communicable chronic disease (NCD), leading to costly complications, reduced life expectancy, and more [1,2]. T2DM and its complications impose a huge economic cost on China, which has the largest diabetes mellitus (DM) burden in the world [3]. Several studies have shown that poor adherence to management behaviors among Chinese T2DM patients resulted in an uncontrolled prognosis of T2DM [4,5,6]. T2DM requires constant management and support to stop the disease from getting worse, which can only be achieved with the coordinated efforts of the patients, families, healthcare institutions, and others [7,8].

These have shown that finding vulnerability factors for managing and developing interventions can help control the deterioration and complications of T2DM in China. With this premise in mind, the Cities Changing Diabetes (CCD) Program was launched to address the social factors and cultural determinants of vulnerability in the management behaviors of T2DM. It is not easy, but it is important to locate the vulnerability factors of T2DM patients. Thematic analysis is used to locate the specific interesting sparks in the text that in our study are the vulnerability factors [9]. Thematic analysis can extract the most salient information from the qualitative interviews. This method also has disadvantages, such as high labor costs, long latency, and humans’ subjectivity, which could cause a bias.

Qualitative interviews which contain a wealth of information such as medical history and treatment methods are appropriate for new-type data mining through Natural Language Processing (NLP) technologies [10]. Pre-trained language representation models such as Bidirectional Encoder Representation from Transformers (BERT) [11] have been used to achieve state of the art performances in most of the tasks related to NLP. The use of specialized language and labels in NLP-based models can greatly improve efficiency when compared to manual classification [12]. Researches on DM using NLP techniques are gradually increasing, and have shown potential in improving the quality of diabetes care [13]. NLP-based diabetes case finding algorithms were shown to identify more DM patients, demonstrating great potential for applications to determine the diagnosis of diabetes more completely [14]. An efficient and accurate phenotyping algorithm was developed and used in the electronic health records (EHRs) to identify DM patients preoperatively, with experimental results confirming its superior performance to other available methods [15]. However, most of the current studies have applied NLP techniques to the diagnosis of DM, and few studies have focused on DM management [13,16].

As the number of patients with DM is rapidly increasing, it is more urgent to fill the knowledge gap on the application of NLP in T2DM management. As the current healthcare reform in China gradually shifts diabetes control to the management level, it is important to help Chinese patients with diabetes to quickly identify vulnerability factors for management behaviors. NLP techniques we used in this study could remedy the labor-intensive, time-consuming, and expensive nature of the traditional thematic analysis. The main objective of this study was to evaluate the applicability of the pre-trained models in this specific Chinese-language medical environment for identifying algorithms to quickly locate vulnerable factors.

## 2. Materials and Methods

### 2.1. Literature Review

Convolutional Neural Network (CNN) [17] achieved the best performance in Chinese medical question intent classification because of the powerful short text feature extraction capability [18]. CNN outperformed the support vector machine (SVM) in a topic classification task for the breast cancer online community [19]. Recurrent Neural Network (RNN) has the ability for building dependencies in neighboring words [20]. A multi-task bi-directional RNN model with the ability to build relationships of dependency in neighboring words was proposed and it performed better than TextCNN in the task of extracting information from Chinese Electronic Medical Records (EMR) [21]. Recently, most text classification in specific Chinese-language medical environments is based on the transformer model, and RNN-based classification models have been less popular.

The transformer is built entirely on a self-attentive mechanism, which not only allows parallel operations but also captures long-range feature dependencies [22]. The emergence of transformer has propelled NLP into the golden age, and BERT and Enhanced Language Representation with Informative Entities (ERNIE) are both products of the transformation of the transformer. BERT, proposed by GOOGLE in 2018, swept the best results of 11 tasks in the NLP domain and took the natural language text classification task to a new level [11]. BERT and its WordPiece tokenization were shown to be superior to the current state-of-the-art models [23]. BERT, a transformer-based model, has been proven to have the ability to classify traditional Chinese medicine (TCM) records effectively [24]. It can make full use of contextual information as a pre-trained model when compared to traditional classification models [11]. ERNIE was the newer model based on BERT, and both the Tsinghua research group and Baidu company chose ERNIE as the pre-trained NLP model name. These ERNIEs have few differences and they all perform well in the tasks with specific Chinese-language medical environments. In the classification task of Chinese eligibility criteria sentences, ERNIE outperformed baseline machine learning algorithms and deep learning algorithms [25].

### 2.2. Bidirectional Encoder Representation from Transformers (BERT)

BERT, which leads the new wave of NLP and even deep learning, uses large unsupervised datasets for language model pre-training and then uses smaller amounts of the labeled datasets for fine-tuning to accomplish specific NLP tasks. Feature-based and fine-tuning are two strategies for applying pre-training language features to tasks [26]. The structure of BERT is a multi-layer bidirectional transformer encoder. For a given token, the input representation is obtained by summing three parts, the corresponding token, segment, and position embeddings.

BERT could fully describe the syntactic-semantic and other information of a text by mining multi-granularity characteristic relations. The BERT model has a bidirectional transformer mechanism that considers the semantic information implied in the context and it can adequately extract features from long and complicated sentences [11,23]. It uses two unsupervised methods, including the Masked Language Model (MLM) and Next Sentence Prediction (NSP), to pre-train together. The former type of MLM can perform random masking on 15% of the words in a sentence, and then use the context to predict the content of the masking. To avoid never seeing certain words in the fine-tuning stage due to a 15% chance of being masked, the developers made 80% of the masked words transposed “[mask]”, 10% unchanged, and 10% randomly replaced with other words. NSP is used to determine the contextual relationship by predicting the coherence of the contextual sentence. BERT is an advanced pre-trained word embedding model based on transformer coding architecture whose resultant output can be one or more vectors [27].

### 2.3. Enhanced Language Representation with Informative Entities (ERNIE)

The model consists of two main layers. The text encoder at the lower level is responsible for capturing the basic vocabulary and information from the input tokens. Another layer is the upper knowledge encoder which is responsible for integrating the knowledge information into the text information to represent the heterogeneous information of tokens and entities into a unified feature space. ERNIE treats a phrase or an entity as a unit which usually consists of several words. During word representation training, all words in the same units are masked instead of just one word or character. ERNIE learns knowledge implicitly without adding knowledge embedding directly, as well as using semantic dependency information more often to guide word embedding learning.

**Figure 1:**
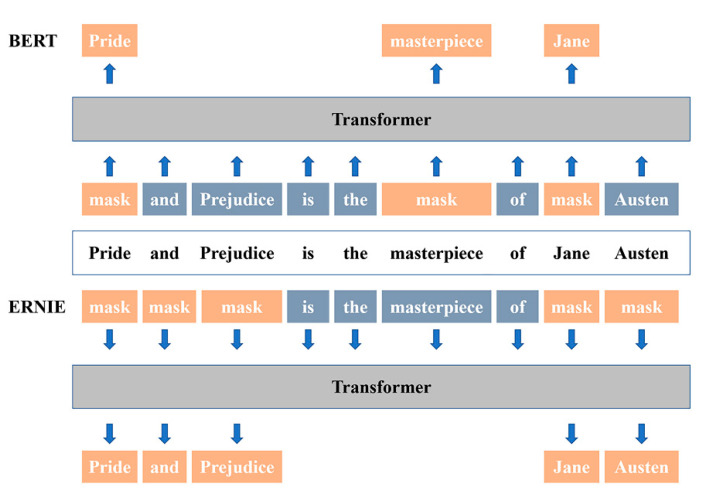
Masking strategy between BERT and ERNIE.

As shown in Figure 1, the masking strategies of the BERT model and the ERNIE model are different. Inspired by the BERT masking strategy, ERNIE was designed to enhance learning language representations through knowledge-masking strategies, including entity-level masking and phrase-level masking [28]. The first learning stage is basic level masking. As with BERT, ERNIE randomly masks 15% of the basic language units and trains the transformer to predict the mask units using the other basic units in the sentence as input. Basic word representations are available at this stage. The second learning stage is the phrase-level masking stage, which is unique to ERNIE. ERNIE uses basic linguistic units as training input, and it masks and predicts all basic units in the same phrase for a random selection of several phrases in the sentence. The third learning stage is the entity-level masking stage. Named entities can be both abstract and actual.

BERT’s masking strategy is based on basic semantic units, which are trained to learn the relationship between words, such as the relationship between PRIDE and MASTERPIECE in the figure above. ERNIE can mask consecutive tokens, not only to learn the word-to-word relationship between BERT but also to learn the knowledge information between PRIDE AND PREJUDICE and JANE AUSTEN. The ERNIE model is more capable of capturing and grasping semantic information. It had shown superior performance over BERT in previous Chinese corpus learning studies [25,29].

### 2.4. Data Source

To make sure we could gain vulnerable participants as much as possible, an inclusion criterion was developed by experts. The case filter and its definition are shown in Table 1. We recruited eligible participants and conducted interviews during August 2015 and March 2016. Finally, this study included 259 participants from 15 hospitals in Tianjin. Field workers recorded semi-structured open-ended interviews with participants. The final collection of 229 interviews was able to meet the requirements for the next step of data analysis. Field workers collected demographic and clinical information about the participants. All demographic information was shown in Table S1. The gender ratio of participants was approximately 1:1, with 106 males and 123 females. The median duration of diabetes of all participants was 13.0 years. 169 (74.1%) participants suffered from complications, and 75 (32.8%) participants suffered from co-morbidities.

Written and verbal informed consent was obtained from all participants. All the methods we used were carried out by the relevant guidelines and regulations. Our experimental protocols were approved by the Ethics Committees of Tianjin Medical University.

After discussing with the CCD Tianjin team and experts from the City University of London (UCL), we developed an initial code manual for thematic analysis. The coder coded two or three interviews according to the coding manual, and then the coder coded a transcript from another member of the team to ensure that the manual coding was valid. During coding, we opened several discussions to perfect the coding manual. Finally, we identified 12 themes and 25 factors associated with patients’ vulnerability. All themes and factors are shown in Table 2.

**Figure 2:**
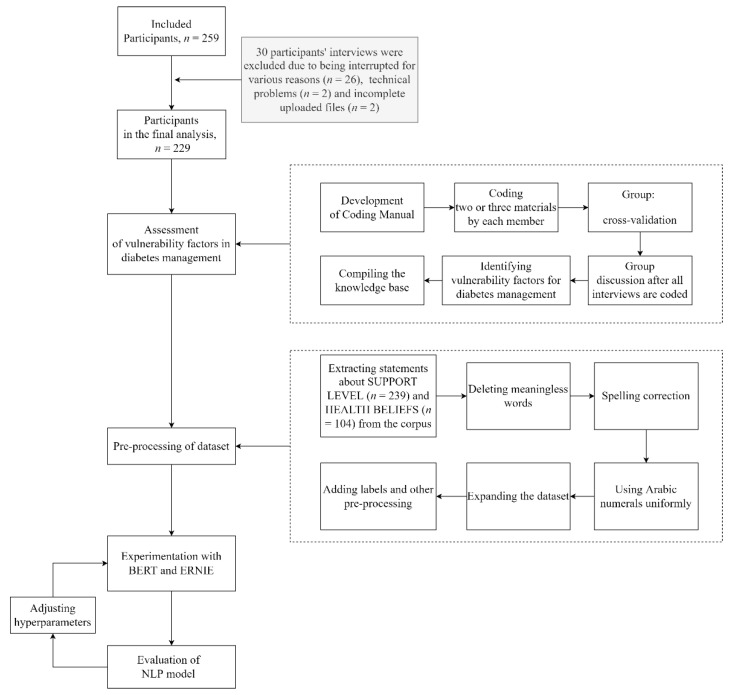
Flowchart of the study.

This research explored the application of BERT and ERNIE in the binary classification of vulnerability factors for DM management. It would be easy to classify texts from very different themes, even with less sophisticated models (e.g., logistic regression). The two themes chosen for this study were HEALTH BELIEFS and SUPPORT LEVEL, which considered the percentage of participants and the similarity of participants’ performance in each theme. HEALTH BELIEFS were mentioned by 92 (40.2%) patients and 212 (92.6%) patients revealed support issues around them. The flowchart of the study is shown in Figure 2.

### 2.5. Pre-Processing of the Dataset

We preprocessed the obtained small corpus manually using the following steps. All operations were carried out manually, and we arranged secondary verification to avoid manual errors.

Deleting Meaningless Words: We removed meaningless words from the sentences, including special expression (operator, unit), stop words, traditional Chinese characters, and full-width characters. We did this to improve the learning efficiency of the model.

Spelling Correction: We corrected the oral words and obvious homophone errors.

Using Arabic Numerals Uniformly: As we all know, the expression form of numbers in Chinese varies a lot. We replaced all numbers appearing in the corpus with Arabic numerals (e.g., three→3, two→2, etc.).

Expanding the Dataset: The dataset was eventually compiled with 343 sentences, which was too small and may affect the pre-training effect of the model. Referring to many researchers who faced similar problems [30], we expanded the number of sentences through operations such as synonymous substitution, changing sentence structure, and sentence transcription. At last, we had 899 sentences about SUPPORT LEVEL and 400 sentences about HEALTH BELIEFS.

Other Pre-Processing: We removed redundant punctuation and added labels after each sentence. The label classified as SUPPORT LEVEL was 0. The label classified as HEALTH BELIEFS was 1. The final 1299 sentences were compiled and stored in a Linux, UTF-8 BOM Txt file. The dataset was randomly divided into the training set, validation set, and testing set by the ratio of 8:1:1, 7:2:1, and 6:3:1.

### 2.6. Experimental Setting

BERT and ERNIE were evaluated by the confusion matrix on the testing set. The special token “[CLS]” was added to express the beginning of the data instance. We added the padding token “[PAD]” to the sentence to process it with a specific length. We converted the criteria sentence into numerical vector by mapping each character to its corresponding unique value [25]. In training, we used cross-entropy loss function and AdamW optimizer in BERT and ERNIE. Finally, a fully connected layer was used to output the classification probability results.

Batch size is the number of samples trained in each batch. Epoch refers to the process of propagating the complete dataset once in the forward and once in the reverse direction through the neural network. In this research, the hyperparameters of both models were set to the same values. Hyperparameters of the ERNIE and BERT were shown in Table 3. BERT and ERNIE are developed based on Python version 3.8.5 (Python Software Foundation, Wilmington, DE, USA). 

### 2.7. Model Evaluation

In this study, we used precision, recall, F1 score, test accuracy, and completion time for comprehensive comparisons of classifier performance. F1 score as a comprehensive evaluation index can reflect the classification of the classifier. The macro-F1 score of the model is the average of the F1 scores of all classes. The results correctly classified by the model are called true positive (*TP*) and true negative (*TN*).

Test Accuracy presents the ratio of correctly classified results to all classified results.

(1) $$Test\;Accuracy=\frac{TP+TN}{P+N}$$

Precision is defined as:

(2) $$Precision=\frac{TP}{TP+FP}$$

Recall is defined as:

(3) $$Recall=\frac{TP}{TP+FN}$$

F1 score is defined as:

(4) $$F1=\frac{2\times Precision\times Recall}{Precision+Recall}$$

To prevent the differences between the F1 scores of various classes from being difficult to distinguish, we calculated macro-F1 scores for comparison.

(5) $$Macro-F1=\frac{F_{\left(HEALTHBELIEFS\right)}+F_{\left(SUPPORTLEVEL\right)}}{2}$$

## 3. Results

In total, the corpus used for the experiments contained 899 sentences about SUPPORT LEVEL and 400 sentences about HEALTH BELIEFS. The detailed data (*n* = 1299) distribution of the two categories for training, validation, and testing is shown in Table 4. For the experiment, hyperparameters of BERT and ERNIE were set as shown in Table 3. We evaluated the applicability of models based on the confusion matrices and performance metrics. Text classification was fundamentally a mapping process, and the ideal state was to point all correct results to the correct set. The confusion matrix and the derived evaluation metrics presented the classification results explicitly. The original data (*n* = 393) distribution of the two categories was shown in Tables S2. Table S3 showed the confusion matrices of the two models with different splitting ratios.

Table 5 showed the test accuracies and completion time of the two models with different splitting ratios. The pre-trained models BERT and ERNIE achieved stable performance under different splitting ratios, and their test accuracies were both above 95%. In general, the completion time of BERT was shorter, and the test accuracy was similar to that of ERNIE. When the batch size of ERNIE was increased from 32 to 64, the completion time was greatly reduced. When the batch size of BERT was adjusted, the test accuracy and completion time changed less.

Table 6 showed the performance metrics of the two models for each class under different conditions. Both categories achieved the F1 score above 0.90 by BERT and ERNIE. The F1 score of HEALTH BELIEFS was 0.9836 when the ERNIE’s batch size was 32 and the splitting ratio was 7:2:1. When the batch size of BERT was 64 and the splitting ratio was 6:3:1, the F1 score of SUPPORT LEVEL was 0.9684. Since ERNIEs with different batch sizes exhibit the same confusion matrix with the splitting ratio of 8:1:1, they had the same values of precision, recall, and F1 score.

Combining the evaluation metrics in Table 5 and Table 6, it was not hard to find the applicability of the pre-trained models in the specific Chinese-language medical environment, where both models performed well and were stable. With the splitting ratio of 8:1:1 and batch size of 64 for ERNIE, the test accuracy was 97.67%, the completion time was 12 min 36 s, and the macro-F1 score was 0.9734. With the splitting ratio of 6:3:1 and batch size of 64 for BERT, the test accuracy was 97.71%, the completion time was 10 min 24 s, and the macro-F1 score was 0.9752. From the application point of view, what we pursued was to obtain the highest performance metrics scores in the shortest time. We considered that BERT performed better in this NLP task with a shorter completion time.

## 4. Discussion

The two themes that were chosen for the binary classification experiment with NLP were HEALTH BELIEFS and SUPPORT LEVEL for several reasons. On one hand, our study was a small-sample study based on a vulnerable population with poor adherence to diabetes management behaviors in Tianjin. In the field of machine learning, acquiring more training data is usually beneficial to improve the accuracy of the model [31,32]. So, we started by picking out the most mentioned theme SUPPORT LEVEL from the interview materials. On the other hand, the more similar the dataset to be classified, the more the classification level of the model can be demonstrated. On this basis, we chose the theme of HEALTH BELIEFS. The main expression of both themes was that individuals were influenced by their families. Family members are considered to be an important part of the support network for patients with diabetes [33]. According to these, we chose two themes for text classification experiments. While the number of sentences for the two themes was imbalanced, this reflected the authenticity of our corpus. Precision is highly sensitive to false positives and is not impacted by a large total real negative denominator. We could see from the evaluation metrics in Table 6 that the Precisions for both categories were above 0.90. The imbalance in the dataset did not have a significant effect on the experiment.

In this study, it took four months to classify the corpus using thematic analysis, while using NLP for topic classification was efficient and saved human resources. The results showed that the completion time of ERNIE with a batch size of 32 was much longer than that of BERT, while there were no differences in test accuracy. According to the literature [34], more powerful GPU would allow for higher batch sizes, which would shorten the total execution time of the training phase. Therefore, this study further investigated the performance comparison of models under different batch sizes, which aimed to shorten the training time by adjusting the batch size. It was obvious that the completion time of ERNIE was significantly reduced by increasing the batch size and the BERT was almost unchanged. At the same time, we also discovered that as the batch size increased, the test accuracy of ERNIE decreased by at least 1.5% for the splitting ratios of 7:2:1 and 6:3:1. Both models achieve macro-F1 scores above 0.95 for different batch sizes and splitting ratios. Our findings demonstrated the applicability of pre-trained models to the corpus of T2DM management vulnerability.

In the classification task of Chinese eligibility criteria sentences, the pre-trained models all performed very well with similar macro-F1 scores, which was consistent with the results of this study [25]. Recent studies have tended to modify the structure of the pre-trained model to obtain higher values of the evaluation metrics. A character-level short text classification model based on BERT, a Robustly Optimized BERT Pretraining Approach (RoBERTa), XLNet, and ERNIE was proposed for eligibility criteria text classification, and they use the focal loss as a loss function to solve the problem of data imbalance among different categories [35]. SMOTE has also been commonly used in some studies to solve the problem of data imbalance by oversampling some minority classes [36]. The above methods brought us insights to solve the problem of data imbalance in the subsequent multicategory study.

It is worth noting that our study has several limitations. First, since the participants were mostly elderly, there might be semantic bias caused by unclear expressions. Second, the low sample size caused the dataset to be too small, and the small dataset greatly affected the effectiveness of the pre-trained model. So, we expanded the dataset to a ratio of about 1:4. The expansion of the dataset was based on the original data, and the low diversity of the data may be the reason for our higher-than-expected results. We will continue to recruit T2DM patients with case filters to expand the corpus for multi-category experiments. Finally, due to the limitations of the GPU, we only explored the performance comparison of the pre-training models with a batch size of 64 and batch size of 32.

The thematic analysis of the interview materials allowed us to be aware of the vulnerability factors in the management behaviors of T2DM patients in Tianjin. Through the application of pre-training models to the CCD corpus, we confirmed the feasibility of NLP techniques in this specific Chinese-language medical environment. Applying NLP techniques to the CCD Program will help to identify how vulnerability factors play out. In the future, we will continue to use case filters to recruit participants to collect interview materials, thus obtaining real statements to expand the dataset. These findings set the stage for identifying barriers and opportunities for successful T2DM management in Tianjin.

## 5. Conclusions

In this paper, a qualitative vulnerability assessment was used to construct 12 themes of vulnerability related to the health and well-being of people with T2DM in Tianjin. A CCD corpus on binary classification was created to explore the applicability of pre-training models in this specific Chinese-language medical environment. Our results showed that BERT performed better in this NLP task with a shorter completion time. Our study provided strong support for using NLP techniques to rapidly locate vulnerability factors in diabetes management.

## Figures and Tables

**Table 1:** Case filter and its definition.

Case Filter	Definition
High BMI	BMI > 28 kg/m^2^
High blood glucose levels	FBG > 6.1 mmol/L, 2 h PBG > 7.8 mmol/L
Duration if diabetes/comorbidities	Have co-morbidities
Health insurance	No worker basic insurance, urban basic insurance or commercial health insurance
Employment status	Unemployment
Below poverty level	For urban residents, the per capita income is less than 705 Yuan, for rural residents, the per capita income is less than 540 Yuan
Body size and physical characteristics	Waistline: male ≥ 90 cm, female ≥ 85 cm
Distance between home and work	≥16 km
Education background	Primary and illiteracy
Physical activity level	Low (civil service, no exercise, etc.)

**Table 2:** Themes and factors of vulnerability of diabetic patients in Tianjin.

Themes	Factors
Financial constraints	Low income
	Unemployment
	No medical insurance/Low reimbursement ratio
	Significant family expenditure
Severity of disease	Appear symptoms, complications, comorbidities Poor disease control
Health literacy	Low literacy
Health beliefs	Perceived diabetes indifferently
	Acquire health knowledge passively
	Distrust of primary health services
Medical environment	Needs not met by medical services
Life restriction	Limited daily life behaviors
	Occupational restriction
Lifestyle change	Adherence to the traditional or unhealthy diet
	Lack of exercise
	Low-quality sleep
Time poverty	Healthcare seeking behaviors were limited by work/taking care of family issues
Mental condition	Appearance of negative emotions towards diabetes treatment or life
Support Level	Lack of community support
	Lack of support from friends and family
	Lack of social support
Social integration	Low degree of social integration
	Faith in suffering alone
Experience of transitions	Diet transformation
	Dwelling Environment/Place of residence transformation

**Table 3:** Hyperparameters of ERNIE and BERT.

Hyperparameters	Value
Hidden Size	768
Learning Rate	5 × 10^−5^
Pad Size	16
Require Improvement	1000
Epoch	100
Batch Size	32

**Table 4:** The detailed data distribution of each category for training, validation, and testing.

Splitting Ratio	Class	Training Set	Validation Set	Testing Set
8:1:1	HEALTH BELIEFS	316 (30.41%)	43 (32.82%)	41 (31.78%)
	SUPPORT LEVEL	723 (79.54%)	88 (67.18%)	88 (68.22%)
7:2:1	HEALTH BELIEFS	282 (31.02%)	78 (30.12%)	40 (30.53%)
	SUPPORT LEVEL	627 (68.98%)	181 (69.88%)	91 (69.47%)
6:3:1	HEALTH BELIEFS	240 (30.81%)	112 (28.79%)	48 (36.64%)
	SUPPORT LEVEL	539 (69.19%)	277 (71.21%)	83 (63.36%)

**Table 5:** Text Acc and completion time of models.

Name	Batch Size	Splitting Ratio	Test Acc (%)	Completion Time
BERT	32	8:1:1	96.12	0:07:38
		7:2:1	96.95	0:07:46
		6:3:1	96.18	0:09:27
	64	8:1:1	97.67	0:09:29
		7:2:1	96.18	0:09:43
		6:3:1	97.71	0:10:24
ERNIE	32	8:1:1	97.67	0:35:36
		7:2:1	97.71	0:48:31
		6:3:1	96.95	1:29:05
	64	8:1:1	97.67	0:12:36
		7:2:1	96.18	0:10:16
		6:3:1	95.42	0:08:45

**Table 6:** Comparison of performance metrics by class.

Name	Batch Size	Splitting Ratio	Class	Precision	Recall	F1	Macro-F1
BERT	32	8:1:1	HEALTH BELIEFS	0.9663	0.9773	0.9718	0.9551
			SUPPORT LEVEL	0.9500	0.9268	0.9383	
		7:2:1	HEALTH BELIEFS	0.9677	0.9890	0.9783	0.9635
			SUPPORT LEVEL	0.9737	0.9250	0.9487	
		6:3:1	HEALTH BELIEFS	0.9432	1.0000	0.9708	0.9580
			SUPPORT LEVEL	1.0000	0.8958	0.9451	
	64	8:1:1	HEALTH BELIEFS	0.9885	0.9773	0.9829	0.9734
			SUPPORT LEVEL	0.9524	0.9756	0.9639	
		7:2:1	HEALTH BELIEFS	0.9886	0.9560	0.9721	0.9560
			SUPPORT LEVEL	0.9070	0.9750	0.9398	
		6:3:1	HEALTH BELIEFS	0.9762	0.9880	0.9820	0.9752
			SUPPORT LEVEL	0.9787	0.9583	0.9684	
ERNIE	32	8:1:1	HEALTH BELIEFS	0.9885	0.9773	0.9829	0.9734
			SUPPORT LEVEL	0.9524	0.9756	0.9639	
		7:2:1	HEALTH BELIEFS	0.9783	0.9890	0.9836	0.9728
			SUPPORT LEVEL	0.9744	0.9500	0.9620	
		6:3:1	HEALTH BELIEFS	0.9759	0.9759	0.9759	0.9671
			SUPPORT LEVEL	0.9583	0.9583	0.9583	
	64	8:1:1	HEALTH BELIEFS	0.9885	0.9773	0.9829	0.9734
			SUPPORT LEVEL	0.9524	0.9756	0.9639	
		7:2:1	HEALTH BELIEFS	0.9574	0.9890	0.9730	0.9541
			SUPPORT LEVEL	0.9730	0.9000	0.9351	
		6:3:1	HEALTH BELIEFS	0.9529	0.9759	0.9643	0.9503
			SUPPORT LEVEL	0.9565	0.9167	0.9362	

## Data Availability

Not applicable.

## Supplementary Materials

The following supporting information can be downloaded at: https://www.mdpi.com/article/10.3390/healthcare10061119/s1, Table S1: The demographic information of all participants; Table S2: The original data distribution of each category; Table S3: The confusion matrices of the two models with different splitting ratios.
