# Supplementary material for: A Comparative Study of Natural Language Processing Algorithms Based on Cities Changing Diabetes Vulnerability Data
Source: Healthcare (Basel). 2022 Jun 15;10(6):1119. doi: 10.3390/healthcare10061119 (PMC9223144; doi:10.3390/healthcare10061119)
Supplement: Supplementary file 1 [file healthcare-10-01119-s001.zip › healthcare-1752720-supplementary.pdf]

Table S1. The demographic information of all participants.

		All participant(n=229)
Age, x±s		56.36±13.36
Sex, *n* (%)		
	Male	106(46.3)
	Female	123(53.7)
Illiteracy or educated primarily, *n* (%)		
	No	7(3.1)
	Yes	7(3.1)
	Missing	215(93.9)
Ever pregnant, *n* (%)		
	No	24(19.7)
	Yes	98(80.3)
	Missing (Except males)	107(46.7)
Number of birth given, *n* (%)		
	1	41(41.8)
	2	22(22.4)
	3	11(11.2)
	4	1(1.0)
	5	1(1.0)
	Missing (Except males)	47(48.0)
Ethnic, *n* (%)		
	Han	221(96.5)
	Hui	5(2.2)
	Others	3(1.3)
Married, *n* (%)		
	Yes	214(94.3)
	No	6(2.6)
	Devoiced	7(3.1)
	Missing	2(0.9)
FBG, *M(IQR)*		8.1(7.0-10.0)
2h PBG, *M(IQR)*		11.2(9.3-14.0)
Glycosylated hemoglobin (%), *M(IQR)*		8.4(7.1-10.0)
Weight, *M(IQR)*		70.0(62.0-80.0)
Height, x±s		166.3±8.3
Waistline, *M(IQR)*		90.0(83.3-98.0)
Abdominal obesity *, *n* (%)		
	No	74(32.3)
	Yes	139(60.7)
	Missing	16(7.0)
BMI, *M(IQR)*		25.9(23.4-28.4)
Complications		
	No	59(25.9)
	Yes	169(74.1)

	All participant(n=229)
Missing	1(0.4)
Type of complications	
Brain	25(14.79)
Kidney	49(28.82)
Eye	64(37.6)
Peripheral nerve	111(65.3)
Heart	70(41.2)
Foot	7(4.1)
Co-morbidities	
No	144(62.9)
Yes	75(32.8)
Missing	10(4.4)
Type of Co-morbidities	
Chronic respiratory disease	8(9.4)
Disable	4(4.7)
Heart and cerebral vessels	63(74.1)
Tumor	7(8.2)
Neuro-degeneration	2(2.4)
Duration of diabetes *M(IQR)*	13.0(1.0-29.0)

* Abdominal obesity: waistline males:≧90cm, female:≧85cm

Table S2. The original data distribution of each category.

Splitting Ratio	category	Training set	Validation set	Testing set
8:1:1	HEALTH BELIEFS	202(84.51%)	22(9.21%)	15(6.28%)
	SUPPORT LEVEL	77(74.04%)	12(11.54%)	15(14.42%)
7:2:1	HEALTH BELIEFS	165(69.04%)	49(20.50%)	25(10.46%)
	SUPPORT LEVEL	79(75.96%)	17(16.34%)	8(7.70%)
6:3:1	HEALTH BELIEFS	145(60.67%)	73(30.54%)	21(8.79%)
	SUPPORT LEVEL	63(60.58%)	32(30.77%)	9(8.65%)

Table S3. The confusion matrices of the two models with different splitting ratios.

Name	Batch Size	Splitting Ratio	True Results	
			**HEALTH BELIEFS**	**SUPPORT LEVEL**
BERT	32	8:1:1	86(97.73%)	38(92.68%)
		7:2:1	90(98.90%)	37(92.50%)
		6:3:1	83(100.00%)	43(89.58%)
	64	8:1:1	86(97.73%)	40(97.56%)
		7:2:1	87(95.60%)	39(97.50%)
		6:3:1	82(98.80%)	46(95.83%)
ERNIE	32	8:1:1	86(97.73%)	40(97.56%)
		7:2:1	90(98.90%)	38(95.00%)
		6:3:1	81(97.59%)	46(95.83%)
	64	8:1:1	86(97.73%)	40(97.56%)
		7:2:1	90(98.90%)	36(90.00%)
		6:3:1	81(97.59%)	44(91.67%)
